# Microfluidic Devices: Useful Tools for Bioprocess Intensification

**DOI:** 10.3390/molecules16108368

**Published:** 2011-09-30

**Authors:** Marco P.C. Marques, Pedro Fernandes

**Affiliations:** 1 Department of Bioengineering, Instituto Superior Técnico (IST), Universidade Técnica de Lisboa, Av. Rovisco Pais, 1049-001 Lisboa, Portugal; 2 IBB-Institute for Biotechnology and Bioengineering, Centre for Biological and Chemical Engineering, IST, Lisboa, Portugal

**Keywords:** microfluidic devices, bioprocess intensification, modeling and simulation

## Abstract

The dawn of the new millennium saw a trend towards the dedicated use of microfluidic devices for process intensification in biotechnology. As the last decade went by, it became evident that this pattern was not a short-lived fad, since the deliverables related to this field of research have been consistently piling-up. The application of process intensification in biotechnology is therefore seemingly catching up with the trend already observed in the chemical engineering area, where the use of microfluidic devices has already been upgraded to production scale. The goal of the present work is therefore to provide an updated overview of the developments centered on the use of microfluidic devices for process intensification in biotechnology. Within such scope, particular focus will be given to different designs, configurations and modes of operation of microreactors, but reference to similar features regarding microfluidic devices in downstream processing will not be overlooked. Engineering considerations and fluid dynamics issues, namely related to the characterization of flow in microchannels, promotion of micromixing and predictive tools, will also be addressed, as well as reflection on the analytics required to take full advantage of the possibilities provided by microfluidic devices in process intensification. Strategies developed to ease the implementation of experimental set-ups anchored in the use of microfluidic devices will be briefly tackled. Finally, realistic considerations on the current advantages and limitation on the use of microfluidic devices for process intensification, as well as prospective near future developments in the field, will be presented.

## 1. Introduction

Biobased processes have been a mainstay of development for thousands of years, but the current trend towards the use of sustainable production methods has further stressed the role that biotechnology can play in the production of a wide array of products and energy [1,2]. Actually, the need for more cost-effective process, with a lower carbon footprint, and less dependence on fossil fuels spans the chemical, pharmaceutical and bio-based industries [1]. Process intensification has been recognized as a sound approach to comply with such requirements [3]. As defined by Charpentier, this methodology encompasses the replacement of large and expensive equipment/processes with cheaper, smaller, more efficient ones. Preferably, it integrates as many operations as possible into a single one, and has been applied to processes in the chemical, pharmaceutical and bio-based industries [1,2]. Miniaturized devices (viz. microreactors, microseparators, micro heat-exchangers), and concomitantly process design based on their application, are among the tools used to implement process intensification [3,4,6,7,8,9]. This is clearly expected, since this trend represents a decrease in size in several orders of magnitude, which results in smaller plants, hence with lower costs in capital and energy, reduced environmental impact, operating in a contained, well controlled and safer environment, where continuous mode of operation is clearly privileged [8,10,11]. Furthermore, process intensification considerably decreases time to market, which is a critical issue in some sectors such as fine chemicals and pharmaceutical industries. Ideally, process intensification allows the direct use of a continuous process developed in lab-scale as the commercial scale process [10]. Since chemical modifications are the core of (bio)chemical processes, miniaturization has focused on microchannel reactors, where at least one dimension is smaller than 1 mm, most likely within tens to hundreds of micrometers, that can be prepared either by microtechnology and precision engineering, or by modification and assembly of microcapillaries. In the former configuration, microchannels are embedded in plates, which can be made of ceramic; glass; polymeric materials (viz. polydimethylsiloxane, PDMS and epoxy-polymer SU-8); stainless steel; and Teflon [12,13,14,15]. These materials can actually also be used for the manufacturing of microcapillaries. The selection of the materials used for the fabrication of the microdevices is naturally influenced by the process conditions envisaged, as summarized in Table 1.

The nature of these microreactors enables operation in microfluidic environment, where only microliter volumes of solution are required [12,14,16]. When addressing bioconversions, the present review will thus focus on microchannel reactors. Other type of microreactors, with operating volumes under 1 mL, which emulate typical bench-scale reactors, have been thoroughly reviewed recently [17], and will not be considered here. Besides their application as reactors, microfluidic devices have also been used for downstream steps, namely involving extraction of large biomolecules, as well as for the integration of (bio)conversion and extraction in a single step [16]. As an outcome of the size reduction concomitant to the use of these miniaturized devices, where surface to volume ratios of 5.0 × 10^4^ m^2^m^−3^ are achievable [11,12,18], significant enhancement in heat and mass transfer is observed, due to the small diffusion path lengths. This allows for nearly gradientless conditions, hence processes take place under a more controlled environment than in conventional set-ups, favoring yield and selectivity [8,19,20,21,22]. Therefore, it is not surprising that the environment in microreactors differs from the one found in conventional scale systems, which is reflected by the effect of increased miniaturization of length scale on transport properties and acting forces, as previously reviewed [11,16,23,24,25]. It has become evident that as a result of downscaling, gravitational and inertial forces tend to lose relevance, whereas viscous and interfacial forces become dominant, while mixing relies mainly on molecular diffusion [26,27]. The small diffusion path has been considered as the major driving force for bioprocess intensification, albeit other process intensification fields/driving forces, such as electric fields, microwaves or pressure can be used. However, although these can be integrated with microreactors for enhancing reaction rates in purely chemical processes, in the case of bioprocesses their integration is more adequate in stages other than the bioconversion/fermentation step [13,28,29,30,31]. 

**Table 1:** A brief overview of how process parameters may condition the selection of materials for the fabrication of microfluidic devices (adapted from [13,15]).

Material	Process variables influencing material selection
Ceramic	Thermal and chemical endurance, but penalized by significant development costs and by shrinkage after sintering
Glass	Ease of visualization and overall chemical endurance but incompatible with strong aqueous bases
Plastic	Low cost and fast fabrication but incompatible with organic solvents and extreme temperature and pressure
Silicone	Compatible with high temperature and pressure and high-aspect ratio design but incompatible with strong aqueous bases
Stainless steel	Compatible with high temperature and pressure but sensitive to corrosive solutions unless expensive metal alloys are used
Teflon	Inertness to several chemicals and extreme resistance against all solvents but relatively unexploited approach

In the two next sections, aspects related to the hydrodynamics in the microfluidic environment and to the configuration of microchannel devices will be addressed, since these establish some boundaries to the practical use of said devices and contribute to provide an up-to-date insight into the current stage of development within the field of microfluidics. Subsequently, the role of microchannel devices within the scope of bioprocess development will be considered. Such matter will be illustrated with some relevant examples. These will help to establish the potential and limitations of microfluidic devices in different stages and of bioprocess development, either in the transformation or the downstream steps. Microchannel reactors provide suitable platforms for screening of biocatalytic activity at intermediate stages of process development, particularly if integrated with spectroscopic tools enabling in-line analysis. Under well controlled conditions, microfluidic devices can constitute representative scaled-down systems, where sets of realistic operational parameters can be evaluated in short time periods. The use of microchannel reactors in production stage through numbering-up can also be considered, the most straightforward example of said use being for bioconversions involving the use of immobilized enzymes. Yet, the implementation of such process requires a careful evaluation against the current conventional large scale approaches used, both considering the performance of the reactors as well as cost issues. 

## 2. Hydrodynamics in Microfluidic Environments

Fluid flow is a key issue in microfluidics on which depend the successful applications of both heat and mass transfer relying in three distinct parameters: (i) channel geometry, (ii) properties of the fluids and (iii) flow conditions [32]. Given the small dimensions of the channels involved, the flow regime is typically laminar, despite reports on transitional regimes [32]. This type of flow favors the control and modelling of bio- chemical reactions while providing high surface area to volume ratio [16]. 

The establishment of stable flow patterns is very important and is determined by the balance of inertial, viscous and interfacial forces [34,35]. Recently it was described that wetting properties (contact angle), influence two-phase flow patterns in microchannels [36,37,38]. Depending on the channel geometry and on the flow rates, different flow regimes in co-current can be observed: bubble flow regime, Taylor flow, annular flow and parallel flow [39]. Various reactor configurations have been studied for contacting and mixing fluids, depending on the specific application, including hydrodynamic focusing, manifold splitting and recombination, T- and Y-junctions, chaotic mixing [32]. Factors like physical properties of the dispersion or hydrodynamics of the flow, important for mass transport and reaction applications, must be taken into account. 

A combination of dimensionless numbers indicates the importance of forces, energies and time scales present at the microscale [23,32,40] and are briefly described in the following text. The Reynolds number (*Re*) represents the ratio of inertial to viscous forces and can be defined by:

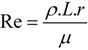
(1)
where *ρ* is the fluid density (kg.m^−3^), *r* is the characteristic velocity (m.s^−1^), *L* is the hydraulic diameter of the microchannel (m) and *μ* is the dynamic viscosity (Pa.s). The dimensionless number that expresses the ratio of gravitational to surface tension forces is the Bond number (*Bo*):

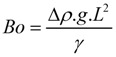
(2)
where *Δρ* is the density difference (kg.m^−3^) and *γ* the interfacial tension between the phase in contact (N.m^−1^), respectively, and *g* is the gravitational acceleration (m.s^−2^). High Bond number indicates that the system is unaltered by surface tension effects while a low number indicates that surface tensions are dominant.

At this scale, typically both numbers (*Bo* and *Re*) are small revealing a control of viscous/interfacial forces over inertial/gravity forces. The Capillary number (*Ca*) expresses the relationship between the two dominating forces (viscous and interfacial) and is defined as:

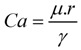
(3)
typically the *Ca* is lower than 1, allowing a distinction between two-phase flow patterns and the mechanism of droplet break-up [41,42]. When *Ca* values become lower than the critical value *Ca_crit_* ≈ 0.1 to 0.01, the surface tension forces break the liquid filament into droplets, minimizing the interracial area. This phenomenon is called the Rayleigh-Plateay instability [43].

Two other parameters based on the Capillary and Reynolds numbers are the Ohnesorge number (*Oh*) and the Weber number (*We*). The Ohnesorge number relates viscous forces to inertial and interfacial tension forces:

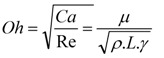
(4)
while the Weber number compares inertial effects to surface tension forces:

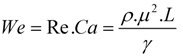
(5)

Despite of the small paths involved, *We* in the range of some hundreds can be obtained in microfluidic devices [44]. Strong surface tensions maintain the droplet as a unique microfluidic entity with a convex interface. If the inertial forces increase, the interface deforms becoming progressively concave and finally disrupting [45,46].

Other dimensionless parameters on fluid properties and operating conditions, include the density ratio (α), viscosity ratio (β) and flow rate ratio (φ):

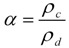
(6)

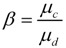
(7)

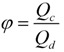
(8)
where *Q* is the fluid flow rate (μL/min^−1^) and *c* and *d* represent the continuous and dispersed phase, respectively, in the case of non-parallel flow [32].

In biotechnology applications a more complex approach is necessary combining not only physical phenomena related to fluid flow but also bio-chemical reaction data. Within this scope several dimensionless numbers take this into account, particularly the Damköhler number [45]. This number verifies if the overall process is limited by the reaction time or by the transport time of the species involved in the reaction:

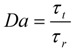
(9)
where τ_r _and τ_t_ are the reaction time and the transport time, respectively [47,48,49]. Within this concept, four Damköhler numbers have been defined [50], the first Damköhler number (Da_I_) considers the relative rates of reaction and convective transport, whereas the second Damköhler number (Da_II_) considers the relative rates of reaction and diffusion [47,51]. On the whole, it is established that for Da largely exceeding unity, transport is the rate limiting step of the overall process, whereas for Da values under 1, the overall process is reaction limited [52]. Swarts and co-workers have evaluated the feasibility of using Da_II_ while establishing the effect of diffusion on enzyme activity in microchannel reactors [53]. These authors reported that, under a specific set of conditions, the value calculated for Da_II_ was different according to the equation used, which could limit its applicability [53]. On the other hand, correlated microreactor performance with Da/K_M_ (Michaelis constant), using as case study a microchannel reactor with a porous wall and reactions with kinetics close to first-order type (based on Michaelis–Menten type) with a low reaction rate [51]. The authors were able to establish that Da/K_M_ have a reduced impact since this ratio could not vary much for the reactions close to first-order type used as reference. 

The Peclet number, Pe, expresses the rate of forced convection to diffusion [45,54]:

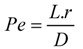
(10)
where a Pe value smaller than 1 reflects dominance of diffusion over convection, whereas a Pe larger than 1 suggests that flow mostly depends on the externally applied driving forces [55]. Since the flow velocities in microfluidic environments are typically small, the channel length is often a critical variable in determining Pe [55]. Given the dimensions used in microfluidic devices, diffusion is dominant, thus Pe is typically small [25]. Occasionally long enough channels may result in Pe values larger than 1, meaning that the forced convection exerted by external forces to create a directed flow of the fluid, is dominant [24,55]. Pe can furthermore allow to establish if Taylor dispersion is relevant or not [55]. Taylor dispersion corresponds to an enhancement of the rate of axial dispersion, due to strong density gradients in the radial direction of flow in microchannels, which exceeds what would be expected due to molecular diffusion alone in the absence of flow [56]. Taylor dispersion can be observed when Pe is considerably smaller than the length to width ratio of the microchannel, whereas diffusion mechanisms hardly contribute to dispersion when Pe largely exceeds the length to width ratio of the microchannel [55]. The performance of microreactors has been compared with that of classical plug flow reactors (PFR) and of perfectly mixed continuous stirred tank reactor (CSTR) [57,58,59]. Although these have been performed using as model systems non-enzymatic reactions, and more specifically systems with pseudo first order and second order kinetics, it has been established that for Pe within 10 and 1, or slightly under, PFR behavior is observed. With a decrease in Pe down to 0.1, a trend towards CSTR is observed, albeit with further decrease of Pe, the performance of the microreactor further shifts from CSTR, and is outperformed by the latter [57,58,59]. 

**Figure 1:**
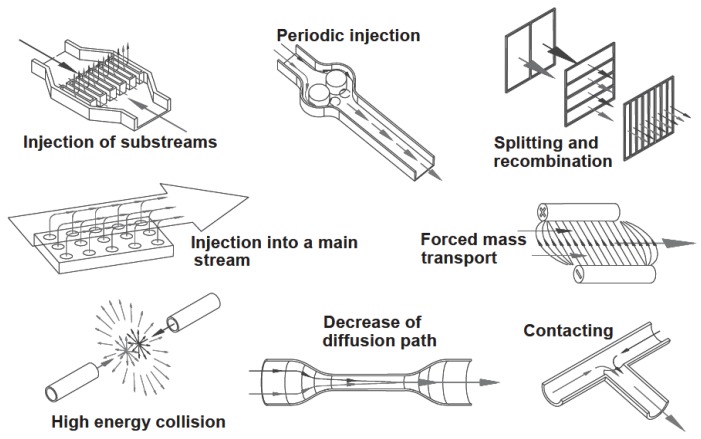
Selective passive and active micromixer principles [Reprinted from [64]. Copyright (2005) with permission from Elsevier].

Mixing depends mainly on the molecular diffusion where mixing time can be defined as:

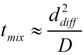
(11)
where d is the characteristic diffusion path (typically the channel width) and *D* is the molecular diffusivity (m^2^.s^−1^). The mixing process by molecular diffusion is slow. A strategy to improve diffusion-induced mixing of reactants is to manipulate the interfacial surface area [60], by using high aspect ratio channels with mixture of different fluid streams accomplished up to tens of seconds, if the channel dimensions are in the hundreds of microns [61,62]. The degree of mixing can actually prove critical regarding product composition for very fast reactions, or when unstable compounds are dealt with [63]. Taking this into account, miniaturized mixers were developed to increase interfacial surface area decreasing consequentially the diffusion length [64,65]. The whole concept of micromixing has actually been thoroughly reviewed recently [40,63,66,67]. Given their small dimensions, and by allowing for a predictable flow pattern, micromixers enable a fast and controllable mixing environment [63]. Besides, micromixers can be used in such a manner that allow for trapping of intermediates (by viz. freeze quenching), and furthermore allow a precise control of reaction conditions by providing a suitable environment for a fast dispersion of added reagents at key time intervals [63]. There exists a great variety of micromixers based on different mixing principles, classified in mainly two basic ways: active and passive micromixers (Figure 1). 

**Figure 2:**
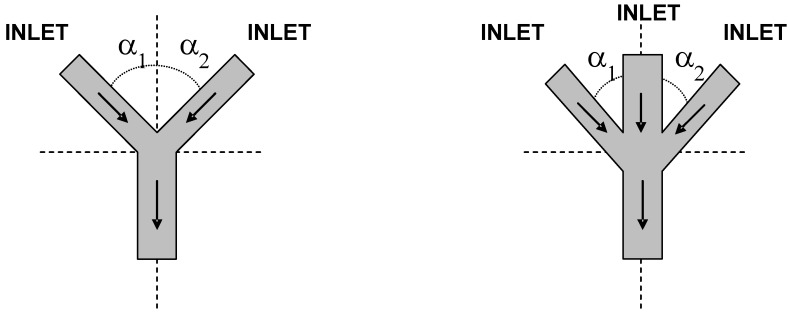
Different ψ- and Y-flow microchannel inlet geometries in multiphase flows. Arrow = flow direction; α - variable that controls the inlet angle responsible for different flow characteristics. In T-shaped inlet, α_1 _= α_2 _= 90°.

In either case, the design aims to decrease the mixing path and enhance the contact surface area [63]. Actually, the range of operation of micormixers in terms of Re and Pe, as well as the mixing efficiency in terms of Re, have been summarized by Kumar and co-workers [40], whereas Lee and co-workers provided thorough data on the performance of micromixers regarding mixing technique, time and length [67]. In active mixing, there is an external energy input are e.g. acoustic, electrical, thermal, pressure disturbance or integrated microvalves/pumps. On the other hand, in passive mixing, there is an induced perturbation on the flow in order to enhance mixing. This is accomplished by e.g., interdigital multi-lamellae arrangements, eddy formation, nozzle injection in flows and collision of jets [62,63,68]. The most common microstructures designs for passive mixing found are zig-zag microchannel, the incorporation of flow obstacle within the channels, T-, ψ- and Y-flow inlet structures and nozzles [23]. The configuration of these some of these inlets is depicted in Figure 2. 

The mixing efficiency allowed by active mixers typically exceeds that of passive mixers, but, on the other hand the fabrication of the former is an expensive and complex process, which has furthermore to comprise the integration of external devices (viz. actuators) into the microreactor. In addition, high temperature gradients may occur in some approaches used for active mixing, which may prove deleterious to biological agents. The whole renders active mixers relatively unpopular when microfluidic applications in biotechnology are considered [63], and will therefore be further considered in this work. Again detailed information can be found elsewhere [63,66]. The classical design of passive mixers relies on T- or Y-shaped microchannells [63,66,68]. In the more straightforward design, mixing relies solely on the diffusion of the species at the interface between the two fluids. Hence, the process is slow and long channels are required. In order to overcome such limitation, and thus enhance mixing efficiency, the microchannels where mixing occurs can be narrowed, hence decreasing the diffusion path; obstacles (*viz* baffles) can be introduced in the channel; the inner surface can be processed in order for the channels to have a rough surface; and operation can be carried out at high Re (viz. over 150) [63,66]. Other designs have been implemented to improve the basic concept, namely [40,63,67]:

a) Multi-lamination, where the inlet stream are divided into several sub-streams, in the form of liquid lamellae, usually within few to several tens of micrometers, which are latter recombined into a laminated stream, the process allowing for enhanced mixing by decreasing diffusion path, while enhancing the contact surface between the two fluids [63,66,69].
b) Hydrodynamic focusing, where three, rather than two inlets are used, resulting in a ψ-shaped format, the inlets being connected to a long microchannel. In this configuration, a solution fed through the middle inlet, flows through the channel, within the outer layer composed by the fluids fed through the side inlets. The flow of the inner fluid is thus constrained, resulting in a thinner lamination width, the length of which depends on the volumetric flow rate ratio between the inner and outer fluids, a larger difference in flow rate resulting in a thinner width, hence favoring mixing [63,66,70,71].
c) Chaotic micromixers, where the transport of a given molecule occurs in a direction transversal to the direction of flow (chaotic advection). As a result of chaotic advection, diffusion flux across interfaces between fluids increases exponentially and striation concomitantly decreases, hence mixing is favored [63,72,73]. Transversal flow can be generated by inserting obstacles in either in the wall of the microchannel or in the microchannel, but with some exceptions [74], this approach is only effective for producing transversal flow when operating at Re over 100 [63]. Alternatively the use of channels with grooved patterns, *viz*. staggered herringbones [40,75], has been suggested as a suitable design to promote chaotic advection, as a result of successions of rotational and extensional local flow, at low Re (*viz*. 1) [63,76,79]. Other approaches for achieving chaotic mixing include serpentine and zigzag flow arrangements [40,67]. In the former configuration, transverse flow in the curved microchannel as an outcome of the consecutive generation of Dean vortices [63,77]. Typically these micromixers are only effective for Re in the range of some hundreds, but improved designs allow effective mixing for low Re [63,78]. In a zig-zag micromixer, transverse flow occurs as a result of recirculation around the turns [79]. Mixing becomes is relatively poor for low Re, and it has been suggested that up to Re around 80, diffusion accounts for mixing [63,67]. The existence of an optimal geometry for this configuration has also been suggested [67].
d) Twisted channels, based on three-dimensional structures of microchannels, with inclined, oblique or wavelike design, where the angle of the bottoms of the channels changes in each subsection. The particular configuration of the microchannel forces the fluid flowing inside to sway around the changeable structures, creating conditions for chaotic mixing to occur, at intermediate Re [67,79,80].
e) Droplet micromixers where the formation of droplets of mixed liquids decreases the path for mixing, as a result of the tree-dimensional internal flow field created by the movement of the droplet, which enables mixing inside the droplet [63,79]. Droplets can be generated as a result of a suitable combination of several factors, namely, significant differences of surface forces (viz. interfacial and viscous) between the fluids, flow in small channels and nature, hydrophobic or hydrophilic of said microchannels, favoring the formation of water-in-oil emulsions or oil-in-water emulsions, respectively. Monodisperse droplets can furthermore be generated, where each of these can be considered an individual reactor, considerably expanding high-throughput capability [63,72,79].

Mixing efficiency can be determined by several flow visualization methods [81] namely *visualization experiments *(aided by microscopic-, photo-, video- or high-speed camera techniques [33,82,83], *reaction experiments* (characterization of mixing with a very fast reaction being a more specific the Villermaux–Dushman [84,85]) and *concentration profiling* (using on- or in-line measurements of optical properties [86]). 

This experimental information is of crucial importance since it validates and develops the numerical techniques to represent the system dynamics. The numerical simulations are anchored mainly on molecular models or on continuum models, depending on the scale of application [87]. The continuum model based on Navier–Stokes equations is used to describe fluid flow when the microchannels are in the range of the micrometers [88,89,90,91,92,93]. To solve the convection term in the Navier–Stokes equations different discretization methods can be applied such as finite element method, finite difference method, finite volume method, or boundary element method [94]. Moreover, to reduce mathematical efforts, and to incorporate data concerning bio-chemical reactions, mass and heat transfer, Computational Fluid Dynamics (CFD) packages have been developed (e.g., Comsol, Ansys Fluent and Ansys CFX) and applied in microtechnology [36,38,95,96,97,98,99,100].

When the length scale of the microchannel reactors are in the region of the nanometer, molecular models are used and can be classified into deterministic (Molecular dynamics [101,102,103]) or statistical approaches (Direct simulation Monte Carlo method [104,105,106,107]), and Lattice-Boltzmann method [108,109,110,111,112,113,114]. 

To aid with the choice of the numerical methods between several factors, a dimensionless number can decide this, namely the Knudson number (*Kn*):

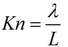
(12)
where *L* represents the physical length path and *λ *the mean free path. At the microscale *Kn* is lower than 1 while at the nanoscale is larger than 1. Typically, for gases *λ* is roughly 1 μm while for liquids, it is smaller, in the range of 5–10 nm [45].

Full description of numerical simulations will not be discussed further here, as this review focuses on giving a general overview on the use of microchannel reactors.

## 3. Configuration of Microchannel Devices

The more common and straightforward configurations of microchannel devices rely either on the assembly of capillary tubes or in the fabrication of a microchannel network on a chip using high precision micromachining techniques, either bulk or surface techniques. In the former, direct modification of the substrate material (*viz*. a monocrystalline silicon wafer, with thickness within several tens to hundreds of micrometers) is carried out, whereas the later is based on the deposition of several layers of thin films of different materials, which are ultimately shaped according to a given design [115,116]. Both surface and bulk micromachining involve a sequence of steps, namely: (a) transfer step, encompassing the growth and deposition of materials; (b) additive step, where etching and removal procedures take place; (c) subtractive step, where different structures and substrates are put together; (d) bonding step, where the shape of the structure is transferred from the template into the substrate by e.g. photo- or nano-imprint lithography or hot embossing [116]. Specific techniques for accomplishing the different steps can vary according to the material to be processed [13]. When microchannel networks are considered two major patterns can be distinguished: (a) a continuous flow microfluidic system, where solutions and solvents are fed by syringe pumps into the continuous-flow microfluidic device through tubing connections , and effluent(s) with the intended product(s) collected at the other end of the device; (b) integrated microfluidics, a more complex system, where a microchannel network is integrated with micromechanical valves and control components. Such assembly allows to perform and to automate complex chemical or biological reactions/processes in a single device. Efforts have thus been made aiming to develop such functioning modules that can improve the performance of microfluidic devices [13,115,117,118]. Among those are mixing modules to overcome diffusion limited mixing typical of the turbulence-free microfluidic environment [13,119,120,121]; pumps enabling the delivery and metering of fluidic components in microchannels [13,122,123]; modules allowing for separation by distillation [124,125,126], by crystallization [127,128,129] or by extraction [130,131]. Integration of modules with different functionalities has been used for analytical purposes [132,133], but also for parallel screening of chemical reactions [121,123,134], for the production of nanowires [135], or for the synthesis of radiopharmaceuticals [115,136] and of oligonucleotides [115,137], and for DNA sequencing [138]. Actually, the potential of integrated microfluidic devices in systems biology, namely within the scope of *omics* processes and *de-novo* synthesis, has been reviewed most recently [139]. Given the nature of their configuration, integrated microfluidic devices can comprehend a set of analytics in the form of a suitable microarray. Such linkage allows one to overcome a typical drawback of bioprocess development typically carried out in continuous-flow microfluidic devices, where often a given microdevice has to be sacrificed in order to provide a suitable aliquot for offline analysis [13,140]. Therefore, efforts have been made in order to integrate sensors for the measurement of physical properties, namely temperature and flow rates, and for monitoring. The later strongly anchor in recent developments in fiber optics and flow-cell technology, which ultimately enable the continuous monitoring of streams in microchannels through several methodologies, among them: attenuated total reflectance-infrared (ATR-IR), near-infrared/ultraviolet/visible (NIR/UV/Vis), Raman and X-ray absorption spectroscopy; nuclear magnetic resonance (NMR); laser-induced fluorescence (LIF). The use of chromatographic methods (HPLC, GC) for monitoring has also been referred to, where samples are transferred from the microreactor to the chromatographic apparatus through suitable combination of micro-syringes and valves [13]. Aiming at the high throughput screening of enzyme inhibitors, using as model the protease cathepsin, de Boer and co-workers integrated a chip-based microreactor with HPLC-electrospray ionization mass spectrometry (ESI-MS). The integrated set-up allowed the simultaneous detection of chemical and biological parameters [141]. Recently, Fang and co-workers integrated a continuous-flow capillary-based microreactor with ultra-high-pressure liquid chromatography (UHPLC) for online analysis, as a set-up for the high-throughput screening system for homogeneous catalyst aimed at an intramolecular Friedel-Crafts addition [142]. An integrated microfluidic system coupling the lysis of cell lines of L929 fibroblasts and of A549 epithelial with an optical fiber, fluorescence-based enzyme assay, was designed to quantify the activity of β-glucocerebrosidase activity, the diagnostic marker for Gaucher’s disease. Enzymatic activity is established based on the cleavage of the synthetic β-glucoside, 4-methylumbelliferyl-β-D-gluco-pyranoside, by monitoring on-line the florescence of the released product, 4-methylumbelliferone, as a function of time [143]. 

## 4. Application of Microstructured Devices to Bioprocesses: Some Examples

### 4.1. Biocatalysis: Overall Considerations

The use of microreactors in biocatalysis can prove beneficial in either process development or at production scale, as recently highlighted [12]. In their realistic appraisal, Bolivar and co-workers suggest that, at the current stage of development, and where process development is concerned, namely at the key stage of screening enzymatic activity [144,145], the throughput available from microtiter plates is higher than that from microstructured reactors. The former configuration is furthermore more advanced when automation and integration with in-line analysis is considered, namely with commercially available systems [12,140,146,147]. Significant developments are taking place within the field of optical sensing systems, (such as microresonator sensing systems), with particular focus on the application to microfluidic devices, which are likely to result in the delivery of commercially available microfluidic devices with in-line analysis capability. The whole can lead to commercially available setups that can compete with the microtiter plate configuration [12,148,149]. Evidence for such pattern is considered to be also provided by the recent reviews on the application of microfluidic lab-on-a-chip platforms [150,151]. While these are developed for diagnostics and specific bio-chemical assays, the strategies developed for incorporating sensors can be used in the microfluidic platforms targeted for bioprocess development. Again, technological developments are increasing the capability of controlled fluid dispensing within microfluidic devices using suitable functional elements (*viz*. microvalves and micropumps) and are furthermore allowing the production of such devices heavily integrated with said functional elements [150]. This can be particularly helpful when screening for enzyme activity, and establishing kinetic parameters, designing multi-step bioconversion systems or optimizing bioconversion/fermentation systems is aimed at. An integrated microfluidic reactor harboring a set of functional elements was effectively tested, using a bovine carbonic anhydrase II (bCAII) click chemistry system as proof-of-concept study, where the synthetic activity of the enzyme over acetylenic benzenesulfonamide and a library of 20 complementary azides was screened. A very similar outcome was observed when the results were matched with those obtained in an experimental setup anchored in conventional 96-well microtiter plates [121]. Jambovane and co-authors fabricated a fully integrated microfluidic chip with sample metering, mixing, and incubation functionalities, and tested the feasibility of the device by establishing kinetic parameters, K_M_ and k_cat_ (turnover number) in a single experiment, and evaluated the effect of inhibitors, phenylethyl β-d-thio-galactoside and lactose, using as model system the hydrolysis of resorufin-β-d-galactopyranoside promoted by β-galactosidase. The authors were also able to report deviations in K_M_ and k_cat_ under 0.3 and 20.4%, respectively, when comparing on-chip and off-chip runs [152]. 

A realistic perspective on the feasibility of process intensification during biocatalytic production scale has again been addressed by Bolivar and co-workers [12]. Taking also as reference data and conclusions compiled regarding chemical transformation, the authors esteem that intensification is a logic step at such stage only if the actual chemical transformation is relatively fast but the overall process is hampered by heat or mass transfer condition. Most biotransformations are relatively slow (with typical rates of k = 0.1–100 s^−1^), suggesting that mass transfer is not limiting, and besides little heat is released. The authors accordingly suggest that significant process intensification at production scale will only occur when considering enzyme catalyzed reactions involving transport across phase boundaries.

Heijnis and co-workers developed a setup anchored in a Y-shaped commercial microchip to study the cross-linking of α-lactalbumin with horseradish peroxidase (HRP) [153]. Hydrogen peroxide, α-lactalbumin and HRP were loaded into the microchannel using a syringe pump and the evolution of the reaction was monitored using a UV-detector connected in-line with the microreactor. The authors were able to develop a suitable reaction model. Furthermore, the bioconversion proved reproducible, judging from the size distribution of the reaction products, which suggests that the setup developed can be used as a fast screening method for the cross-linking of proteins promoted by HRP.

### 4.2 Specific Examples

#### 4.2.1. Immobilized Enzyme Microreactors

Microreactors have been used as scale-down system for complex enzymatic synthesis, such as multienzyme catalysis, cascade reactions, rapid characterization of bioconversion processes and design of microfluidic biofuel cells [12,153,154,155,156]. The application of microstructured reactors in such bioconversion processes is often associated with the use of immobilized enzymes. Immobilization is typically implemented by coating the wall of the microreactor [157], or by packing the microchannels with either small beads or with a monolith structure. The latter structure comprises open channels, hence providing an alternative to the use of microreactors packed with small beads, where large pressure drop can be eventually associated with continuous operation [12,158,159]. Immobilization of enzymes in the surface of channels overcomes backpressure limitations and provides a large interfacial area per unit volume [160], albeit eventually at the cost of enzyme loading. Some recent illustrative examples of the application of these two different approaches are given in Table 2 and Table 3. 

A configuration that eludes the two aforementioned approaches, while still involving packing of a microfluidic reactor has been has been suggested by Schilke and co-workers [161]. It is based in an IR flow cell packed with an enzyme immobilized in silicon dioxide nanosprings in a mat format. Nanosprings are claimed to provide high solvent-accessible surface area, present adequate permeability and mechanical stability, and can be patterned into existing microdevices. The selected enzyme, β-galactosidase, was immobilized in a nanospring mat (2.2 cm^2^ × 60 µm thick) after treating the inorganic support with γ-aminopropyltriethoxysilane, then with N-succinimidyl-3-(2-pyridyl-dithio)-propionate (SPDP), and finally with dithiothreitol, to produce surface thiol groups. Once modified by treatment with SPDT, in order to introduce thiol-reactive pyridyl disulfide groups, the enzyme was covalently bound to the support by a covalent disulfide bond. The nanospring mat biocatalyst was then was placed into a 175-µm high microchannel, and used to study the kinetics and steady-state conversion pattern of the hydrolysis of o-nitrophenyl β-d-galactosylpyranoside under different substrate flow rates and concentrations. The authors were able to produce a numerical model to fit the experimental data and to simulate reactor performance. Furthermore, it is suggested that *in-situ* regeneration by reduction with dithiothreitol followed by incubation with the modified β-galactosidase is possible.

Another alternative configuration for an immobilized enzyme microreactor was developed by Alam and co-workers, combining a microreactor coupled to a cellulose membrane unit, which has been used for the hydrolysis of sugar beet pectin by pectin lyase [162]. In the set-up, which however involves the use of a non-structured reactor, for the latter is of microchemostat-like configuration [12,17,163], process integration is implemented since the membrane unit allows *in-situ* separation of products from unreacted substrate and enzyme. Comparable results were obtained when the continuous membrane microbioreactor and a lab-scale set-up were matched, illustrating the validity of the approach.

Considering the flexibility of processes anchored in the use of microreactors, along the costs of the microstructured devices, a particular challenging issue lies in the development of immobilization strategies that would allow for full removal of the enzyme (or cell) from the immobilization matrix when the catalytic activity decreases or a new enzyme is to be tested. Without prejudice of the stability of the attachment during operation, removal should be easily performed under elution condition. The successful accomplishment of such goal would allow the reuse of microdevices. Widely applicable strategies for reversible protein binding are therefore been looking for, preferably based on the use of specifically tagged proteins [12]. 

Microfluidic reactors have also been used in multi-fluid phase systems, namely for processes involving sparingly water soluble molecules. Work on such systems has given valuable insight on the relevance of several features for the development of a efficient setup, such as nature and ratio of the immiscible phase, or the effect of the chemical composition of the channel surface on phase separation [12,16,154]. Phase separation can be typically achieved taking advantage of small interfacial areas that provide enough capillary pressure to compensate the imposed driving pressure or by modifying the wetting characteristics in order to stabilize the interfaces [177,178]. Since chemically modified surfaces can degrade under process conditions, methodologies for maintaining phase separation by incorporating specifically design and fabricated materials with different surface properties, viz. hydrophilic glass combined with Teflon, were developed [177,178]. Several applications in biocatalysis involving multi-fluid systems rely on the use of free enzymes, since the latter are preferably retained in one of the phases, a feature that already configures an immobilization pattern, ultimately enabling enzyme recovery and re-use. Some relatively recent representative examples are given in Table 4. Nevertheless coupling enzyme immobilization to two-liquid phase systems has also been implemented. Enzyme immobilization in macroporous silica-monoliths with controllable porosity, packed within a capillary column, has been coupled to operation in biphasic liquid systems. An example is the hydrolysis of 4-nitrophenyl butyrate in water–decane media with immobilized *Candida antarctica* lipase A [179]. The kinetic studies performed with showed that k_cat_ was similar to that for free lipase in solution, whereas the apparent K_M_ for the immobilized enzyme was 12-fold lower than that for the free form. Again, a 96 % conversion yield was obtained with the immobilized form, roughly exceeding 4-fold the yield with the free lipase, a feature ascribed to the favorable biphasic system in the continuous flowing micro-reactor system, given the relevant increase in the interfacial activation. Immobilization also enhanced operational stability. Bioconversion systems developed in two-liquid phase systems in microreactor environment are typically performed in co-flow mode. 

Given the growing use of microreactors in applied biocatalysis, attempts have been made to establish a rationale for the design of representative experimental setups anchored in said devices. Using as model system the hydrolysis of *o*-nitrophenyl-β-d-galactopyranoside by β-galactosidase, Swarts and co-authors were able to establish that, for given residence times, diffusion could affect reaction rate [53]. These authors were furthermore able to correlate such critical residence time with operational parameters, namely enzyme concentration, substrate concentration and channel width, therefore contributing for the definition of key issues required for the design of robust and reproducible microreactor system.

Recently, further guidelines have been suggested to optimize the design of co-flow enzyme microreactors [89]. Since mass transfer restrictions can affect reaction rate and productivity, depending on enzyme properties, operation conditions and dimensions of the microreactor, correlations for these later parameters were developed, assuming Michaelis Menten type kinetics. The authors conclude that effectiveness, defined as the ratio of the observed reaction rate to the reaction rate, is a key parameter for microreactor design, but may not lead to optimized throughput, this depending on the configuration of the microreactor. Actually, the effectiveness decreases as function of the channel width, accordingly smaller microchannels minimize mass transfer restrictions. On the other hand, the throughput of co-flow microreactors displays an optimum as function of the channel width, under mass transfer limiting conditions. Maximum throughput and high effectiveness are therefore considered to establish a window of opportunity for the design of co-flow enzymatic microreactors.

**Table 2:** Packed-bed type microreactors: some case-studies.

Enzyme	Immobilization method and reactor	Comments on the immobilized-based system	Reference
(+)-γ-lactamase	Enzyme cross-linking combined with controlled pore glass (1:1) packed in silica-fritted capillary tubes	Hydrolysis of amides. Evaluation of enzyme stability, activity, kinetics and substrate specificity.	[164]
		High activity retention; similar substrate specificity for most substrates and increased for acrylamide as compared to free form	
		Enhanced thermal stability, thus allowing extensive screening tasks using a single microreactor	
Lipase	Novozyme® 435 (enzyme adsorbed on crosslinked PMMA resin, Lewatit VP OC 1600) and packed in glass capillary columns	Chemo-enzymatic epoxidation of olefins was proved feasible, with significant reduction in reaction time as compared to operation in standard batch reactor	[165,166]
		The potential of this approach as a suitable tool for the study of the reaction was established	
Lipase	Novozyme® 435 (enzyme adsorbed on crosslinked PMMA resin, Lewatit VP OC 1600) and packed in microchannels milled on aluminum	Polymerization of ε-caprolactone to polycaprolactone	[167]
		Operation with the microreactor allowed faster polymerization and higher molecular mass of product when compared to operation with batch reactors	
		Corroborates the potential of these platforms for high throughput screening of enzymes and process conditions	
l-aminoacylase	Enzyme immobilization through the reaction of the primary amine groups with epoxy terminal groups on the surface of PGMCED monoliths formed inside the microreactor channels	The authors established the use of the immobilized microreactor as a reliable screening tool for enzyme selectivity, aiming at the production of l -amino acids and their analogues	[168]
		Additionally, the order of preferred N-protecting group (benzoyl) and the order of preferred N-benzoyl protected amino acids were established	
		High thermal and operational stability, allowing the use small amounts of organic solvents and temperatures as high as 50 ºC for bioconversions where substrate solubility could be a limitation	
Glucose oxidase (GOD) and choline oxidase (CHO)	Each enzyme immobilized in sol-gel monolith, where the precursors were allowed to polymerize in the microchannel of a glass microreactor. The monolith was activated with PEI, and the enzyme immobilized through electrostatic interaction between electronegative enzymes and electropositive PEI polymers	Oxidation of glucose (GOD) and choline (CHO)	[159]
		Hydrogen peroxide formed as product of either reaction was quantified amperometrically using an on-chip electrochemical cellData for GOD - k_cat_ similar to that for GOD in solution, albeit with V_max_ (maximum reaction rate) 70 fold higher, and K_M_ 4 fold lower - suggested favorable enzyme concentration in the microenvironment of the monolith and enhanced maintenance of enzyme conformationData for CHO - k_cat_ and K_M_ values similar to those for CHO in solution, but a 50 fold increase in V_max_ – corroborate the efficiency of the methodology	
		Regeneration of the microreactor by rinsing with 1 M NaOH solution, making the operation of the microreactor highly flexible	
Horseradish peroxidase (HRP)	Immobilization onto the surface of gold, with thickness from nano- to micro-scale deposited in a silicon wafers. Binding by adsorption or by covalent interaction with the gold surface	Enzymatic oxidation of phenols	[169]
		The stabilizing effect of immobilization on enzyme activity, the screening capability and the operational stability of the device were established.	
		Regeneration of the microreactor through an electrochemical cleaning procedure, making the operation of the microreactor highly flexible	
Hydroxylaminobenzene mutase and soybean peroxidase	Each enzyme entrapped in silica nanoparticles, which were packed in microfluidic chips.	Chemo-enzymatic synthesis of APO, aminophenoxazin-3-one, from nitrobenzene, by connecting in series three individual microreactors, harboring zinc, mutase and peroxidase.	[170]
		The potential of microfluidic reactors for performing chemo-enzymatic multistep reactions was established	

PMMA: poly-(methyl methacrylate; PGMCED: poly(glycidylmethacrylate-co-ethylenedimethacrylate); PEI: polyethylenimine

**Table 3:** Coated-wall type microreactor: some case studies.

Enzyme	Immobilization method and reactor	Comments on the immobilized-based system	Reference
β-glycosidase	Covalent binding to the surface-activated walls of the stainless steel microreactor	The setup was used for the continuous hydrolysis of lactose	[171]
		Conversion yield in excess of 70%, a space time yield 500 mg glucose mL^-1^ h^-1^, and a half life of 15 days were observed.	
		Results suggest that the immobilized microreactor is a suitable tool for screening, reaction optimization and preparative synthesis on demand	
β-glycosidase (thermostable)	Covalent binding to the surface-activated walls of the stainless steel microreactor	Synthesis of β-glucosylglycerol from cellobiose and glycerol	[172]
		Under selected operational conditions, 120 mM of β-glucosylglycerol were obtained from 250 mM cellobiose and 1 M glycerol	
		Conversion behavior similar to that in a batch stirred reactor with soluble enzyme	
		Rapid identification of suitable reaction conditions, corroborating the high-throughput nature of microreactor for process characterization	
Fumarase	Covalent binding to the inner surface of glass microchannels after silanization with APTES and cross-linking with glutraldehyde	Hydration of fumaric acid to l -malic acid	[173]
		The immobilized enzyme retained 25% of the activity of the free fom, which the authors claimed to exceed previous reported data	
		Selected operational conditions allowed a conversion yield of up to 80%.	
		Development of a predictive 3D model comprising mass transfer and reaction kinetics	
Lipase	Adsorption of lipase on mesoporous silica (MPS) thin film deposited on its inner walls of micro-capillary borosilicate tubes	Enantioselective transesterification of vinyl acetate with (±)-1-phenylethanol	[160,174]
		A 3D cubic structure film allowed a yield of 64%, for an enantioselectivity in excess of 99% in continuous flow experiment	
		The catalytic activity of the immobilized PS exceeded that of the native enzyme	
		High operational stability	
Lipase	Covalent binding to the inner surface of silica microstructured optical fiber after silanization with APTES and cross-linking with glutraldehyde. Microreactors were 20 cm long	Synthesis of butyl laurate from *n*-butanol and lauric acid at 50 ºC, with *n*-hexane and *n* -heptane as solvents.	[175]
		A 3:1 *n* -butanol-lauric mole ratio yield of up to 99% in less than 38 s of residence time.	
		Bioconversion pattern was roughly similar with either solvent, but *n* -heptane may be preferred due to lower toxicity	
		High operational and storage stability	
		Full conversion foreseeable in longer microreactors, which s could pave the way for scaling production by numbering up	
Yeast cells	Covalent binding to the inner surface of microchannels after silanization with APTES and cross-linking with glutraldehyde	Sulfuric acid was shown to be the most effective for surface activation of different materials, namely glass, FEP, PFA, PS and PTFE, prior to silanization with APTES	[176]
		A cell coverage of about 70% was reported in all materials tested	

APTES: 3-aminopropyl-triethoxysilane; FEP: luorinated ethylene propylene; PFA: perfluoroalkoxy; PS: polystyrene; PTFE: polytetrafluoroethylene

**Table 4:** Two-liquid phase bioconversion systems in microreactors: some case studies.

Enzyme	Fluid system and reactor	Comments on the micro-scale bioconversion system	Reference
Cholesterol oxidase	Aqueous phase containing enzyme solution; n-heptane phase containing the substrate. These were separately fed through a Y-shaped inflow to the microchannel of the glass microchip reactor.	Oxidation of cholesterol to 4-cholestene-3-one	[93]
		Selected ratio of the fluid flow rates allowed for phase separation in Y-shaped outflow of the microreactor, enabling *in-situ* recovery of the product (present in the organic phase)	
		Roughly 70% conversion of 0.17 mM cholesterol was obtained for residence times close to 1 minute	
		Characterization of the bioconversion system through a 3D mathematical model comprising mass transfer, kinetics and velocity profiles	
Hydroxynitrile lyase	Aqueous phase containing crude enzyme lysates and HCN; organic phase containing a selected aldehyde. These were separately fed through a Y-shaped inflow to the microchannel of the glass microchip reactor. The product was recovered from the single outflow	Enantioselective synthesis of cyanohydrins from aldehydes	[180]
		Clogging during addition of the lysates to the microchannels did not occur	
		Undefined plug flow was observed inside the microchannel rather than laminar flow, possibly due to detergents or other surfactants present in the cell lysate. Conversion yields over 90% and enantioselectivity in excess of 99% were obtained	
		Results consistent with those from large, batchwise process, validating the microscale approach	
Laccase	l-DOPA and laccase solutions, both in phosphate buffer, were fed from each inflow of the Y-shaped glass microchip reactor	Oxidation of l -DOPA	[181]
		Up to 87% conversion of 0.5 mM of L-DOPA observed at residence times of 100 s	
		Increasing the inlet concentration of L-DOPA decreased conversion efficiency, possibly to the low molecular diffusivity of laccase in water. A longer microchannel could overcome this drawback	
		Characterization of the bioconversion system through a 2D mathematical model considering convection and diffusion, and kinetics	
Lipase	Aqueous phase containing enzyme solution: n-hexane phase containing substrates. These were separately fed through a Y-shaped inflow to the microchannel of the glass microchip reactor.	Synthesis of isoamyl acetate from isoamyl alcohol and acetic acid	[182]
		Selected ratio of the fluid flow rates allowed for phase separation in Y-shaped outflow of the microreactor, enabling *in-situ* recovery of the product (present in the organic phase)	
		Up to 35% conversion for 0.5 M acetic acid and isoamyl alcohol concentrations and residence time 36.5 s, at 45 ºC, superior to those found in the literature, that far	
		Characterization of the bioconversion system through a 3D mathematical model comprising mass transfer, kinetics and velocity profiles	
Lipase	Aqueous phase containing enzyme solution; *n*-decane phase containing substrates. These were separately fed through a Y-shaped inflow to the microchannel of the microchip reactor. The product was recovered from the single outflow after centrifugation	Synthesis of butyl-propionate from the esterification of propionic acid and 1-butanol. The product partitions preferably to the organic phase, while substrates favor the aqueous phase, the whole preventing the reaction to reach equilibrium	[183]
		A Ping Pong Bi Bi mechanism with alcohol inhibition was developed to describe the reaction	
		Kinetic parameters and thermal activation and inactivation patterns matched those observed in bench scale run	
		Validates microfluidic approach for characterization of these systems with evident cost optimization	
Lipase	IL ([bmpyr][dca]) phase, containing lipase, isoamyl alcohol; IL ([bmpyr][dca]) phase containing acetic anhydride; *n*-heptane. These were separately fed through a ψ-shaped inflow to the microchannel of the microchip reactor.	Synthesis of isoamyl acetate from isoamyl alcohol and acetic anhydride	[184]
		A roughly 3-fold increase in the reaction rate was observed for the synthesis performed in microreactor environment, as compared to that observed in a stirred batch reactor, resulting in better productivity than any reported that far.	
		Results were ascribed to the reaction–diffusion dynamics in the microchannel system, enabling an emulsification that led to a large interfacial area for the reaction and simultaneous product extraction.	

l-DOPA: 3,4-dihydroxy-l-phenylalanine; IL: ionic liquid; [bmpyr][dca]: 1-butyl-3-methylpyridinium dicyanamide

#### 4.2.2. Downstream Processing

The technological developments that have been taking place at the level of design and fabrication in micro and nanofluidics environments, namely at the level of microfluidic handling liquid, *viz*. micromixers, micropumps, and microvalves, have led to microfluidic platforms that can comprise a wide set of unit operations. Each unit operation corresponds to a building block of laboratory protocol and encompasses fluid transport, fluid metering, fluid mixing, valving, separation or concentration of molecules or particles [150]. The integration of such unit operation in microfluidic platforms has been gaining relevance at the level of devices for point of care diagnostics and for detection and screening of bacteria and drugs, but application of the concept in further areas is gradually being implemented [150,185]. When bioprocess development is considered, most of the applications encompassing the use of microfluidic devices have been centered in the recovery of macromolecules, through liquid-liquid extraction, but also through chromatography. Applications on extractive two-liquid phase systems in microfluidic environment have focused mainly on the recovery and purification of proteins with therapeutic applications, since the intensification resulting from miniaturization decreases the risks of delays during bioprocess development and product launch [186,187]. Some examples of recent applications are given in Table 5. 

Microfluidic chromatography columns have also been developed, aiming at improving the separation of biopharmaceuticals. Shapiro and co-workers used a 1.5 µL volume column packed with different fillings, *viz.* porous agarose beads, Q Sepharose Fast Flow, and were able to establish breakthrough and elution curves while processing different proteins. The authors were able to establish the reproducibility of the data throughout different scales, up to 30 mL laboratory [188,189].

## 5. Conclusions

The use of microstructured reactors within the scope of bioprocess intensification has been gaining momentum, namely due to the advantages brought by the enhanced heat and mass transfer, flexibility, ease of operation under controlled hydrodynamic conditions and low requirements of reagents. Technological developments are contributing for the development of microfluidic platforms, which integrate monitoring and fluid handling functionalities that further expand the range of application of microfluidic devices. Still, the contribution of these devices at bioprocess production scale is scarce, and a realistic appraisal on the feasibility of their application within such scope is undergoing. Further expected developments on this field can be related to the development of enzyme/whole cell immobilization strategies that will allow the reuse of the microfluidic platforms and further increase its flexibility as screening and process characterization tools. Furthermore, guidelines for the design of microfluidic devices as representative systems are being developed.

**Table 5:** Application of microfluidic devices in downstream processing: some examples.

Microfluidic device	Application and comments	Reference
Microchip with ψ-shaped inflow and Y-shaped outflow, for independent feeding of three different solutions and recovery of two separate phases	Isolation of fluorescent, genetically tagged proteins from *E. coli* lysates, through extraction in ATPS (PEG/salt)	[190]
	Laminar flow and low interfacial tension led to a stable interface along the microchannel. The protein was recovered in the PEG rich phase in one of the outflows; contaminants and interphase were recovered in the second outflow.	
	The fluorescent nature of tagged proteins eased the visualization of the extraction process. Roughly 85% of contaminating proteins, unwanted nucleic acids and cell debris, were removed.	
Microchip with ψ-shaped inflow and outflow, for independent feeding of three different solutions and recovery of three separate phases	Purification of bacteriorhodopsin from *Halobacterium salinarium* cells extracts	[191]
	ATPS (PEG/salt) and IL (HHMM/salt) system were compared for protein isolation; cell suspension fed through central inlet, with a three phase flow maintained throughout the microchannel in both ATPS and IL system	
	Contaminants were removed to the PEG (or IL) and salt phases.	
	The recovery rate of protein was roughly similar for both methods, roughly 90%, with purity of 95%, but IL system proved more sensitive to variations in pH, as reflected by the concomitant decrease in the recovery rate	
Microchip with ψ-shaped inflow and outflow, for independent feeding of three different solutions and recovery of three separate phases	Purification of membrane proteins from crude cell through extraction in ATPS (PEG/detergent)	[192]
	Continuous operation in microfluidic environment is clamed to result in increased extraction rate and efficiency when compared to the traditional discontinuous approach	
Microchip with ψ-shaped inflow and outflow, for independent feeding of three different solutions and recovery of three separate phases	Discrimination of live and dead cells from animal cell cultures, through extraction in ATPS (PEG/dextran)	[193]
	Optimized flow rates led to stable aqueous two-phase flows along the microchannel	
	Live recovered in the PEG phase. The recovery efficiency of live cell was up to 97 %, as compared to only 85.5% in the normal macroscale ATPS	
Microchip with ψ-shaped inflow and outflow, for independent feeding of three different solutions and recovery of three separate phases	Use of ATPS (PEG/dextran) for the outflow microchip were used for the separation of leukocyte and erythrocytes from whole blood cells, and for the concentration of leukocytes	[194,195]
Microchip with Y-shaped inflow and outflow, for independent feeding of aqueous and organic phases	Extraction of progesterone and 11α-hydroxyprogesterone from an aqueous phase with ethyl acetate. Model system and integration with a whole cell bioconversion where 11α-hydroxylation is performed by *Rhizopus nigricans* pellets	[196,197]
	Extraction occurred in few seconds and the mathematical model of the extraction developed was shown to correlate with experimental data	
	Further optimization of the extraction and numbering up of microdevices, is likely to result in a realistic integrated system for the production of 11α-hydroxyprogesterone	
Microchip with Y-shaped inflow and outflow, for independent feeding of aqueous and organic phases	Enantioselective separation of racemic amino acids. The model systems integrates the enantioselective deacetylation of N-acetyl- d,l-amino acids in aqueous media carried out in a microreactor; the product phase is acidified andfed to the microchip extractor, where the acetyl-d -amino acid is extracted into the organic phase	[198]
	In most cases, the optical resolution of acetyl- d -amino acid exceeded 98% and the final recovery of an acetyl- d -amino acid from the aqueous phase exceeded 85%	

ATPS: aqueous two phase system; HHMM: hexafluorophosphate (1-n-hexyl-3-methylimidazolium); IL: ionic liquid; PEG: polyethylene glycol
